# Aptamers for CD Antigens: From Cell Profiling to Activity Modulation

**DOI:** 10.1016/j.omtn.2016.12.002

**Published:** 2016-12-10

**Authors:** Amin Nozari, Maxim V. Berezovski

**Affiliations:** 1 Department of Chemistry and Biomolecular Sciences, University of Ottawa, Ottawa, ON K1N6N5, Canada

**Keywords:** aptamers, cluster of differentiation, siRNA, cell imaging, drug delivery

## Abstract

Nucleic acid-based aptamers are considered to be a promising alternative to antibodies because of their strong and specific binding to diverse targets, fast and inexpensive chemical synthesis, and easy labeling with a fluorescent dye or therapeutic agent. Cluster of differentiation (CD) proteins are among the most popular antigens for aptamers on the cell surface. These anti-CD aptamers could be used in cell biology and biomedicine, from simple cell phenotyping by flow cytometry or fluorescent microscopy to diagnosis and treatment of HIV/AIDS to cancer and immune therapies. The unique feature of aptamers is that they can act simultaneously as an agonist and antagonist of CD receptors depending on a degree of aptamer oligomerization. Aptamers can also deliver small interfering RNA to silence vital genes in CD-positive cells. In this review, we summarize nucleic acid sequences of anti-CD aptamers and their use, which have been validated in multiple studies.

## Main Text

### Introduction

Cluster of differentiation (CD) nomenclature was first used by the Human Leucocyte Differentiation Antigens (HLDA) Workshop in 1982 for the characterization and study of leucocyte surface molecules and antibodies against them.^1^ To date, the HLDA (which is now called the HCDM) has approved more than 400 CD designations.^2^ Since the demonstration that, under the right conditions, antibodies can take the place of the natural ligands in cells’ physiological processes, CD antigens have been targeted to manipulate immune responses.^3^ Many anti-CD antibodies have been shown to be efficient in immunosuppression,^4^ cancer therapy,^5^ and transplant tolerance.^6^ Additionally, CD molecules have a vast amount of applications in cell phenotyping and disease diagnosis.^7^ Some CD molecules are better known than others because of their pivotal role in immune system homeostasis, such as CD4, CD8, CD3, CD28, CD45, and CD80.^8^ There are also some CD antigens that play roles in disease incidence, such as CD4 and CCR5 (CD195), which are used by HIV particles as receptors for cell entry.^9^ Enough has been said about the importance of CD antigens, and the critical applications of their antibodies are becoming more obvious. Currently, very few studies have not used anti-CD antibodies as a material or subject, particularly when it comes to immune-related studies, from vaccine development to cancer therapy.

Finding the appropriate antibodies for immune manipulation has always been a challenge. The process of developing the right antibody is very laborious and expensive.^10^ Many antibodies that show promising results in in vitro and animal studies never come to market due to the many complications of their production process, including a low yield of production, toxicity, low bioavailability, and high immunogenicity.^10^ The alarming need for a new alternative is now felt more than ever before. It has been suggested that nucleic acid-based aptamers might be the answer. Aptamers are small, single-stranded oligonucleotides that show significant affinity toward their targets, usually with high specificity and sensitivity.^11^ The advantages of using aptamers over antibodies have been mentioned in many studies, and here, we do not intend to iterate and instead refer you to other good reviews of this matter.^12^, ^13^, ^14^, ^15^

Recently, multiple aptamers have been developed that specifically target CD molecules. It has been shown that aptamers can act as an inducer or an inhibitor of signaling pathways.^16^ Moreover, it was recently shown that aptamers can act simultaneously as an agonist and an antagonist, a feature that is unique compared to antibodies.^16^, ^17^ This ability of aptamers to easily trigger different responses through their ligand binding makes them very good candidates for the development of CD molecules for the purpose of manipulating immune responses. During the last few decades, aptamers have been found for some CD antigens, and in recent years, they have caught the interest of scientists because of their promising applications. In this review, we will take a look to these aptamers and their new applications, and we will discuss the future of the aptamer field, with a focus on CD antigens. All aptamer sequences mentioned in the article are presented in Table 1.

### From Cell Staining to Prophylaxis for HIV: CD4 and CD195 Aptamers

**Figure 1:**
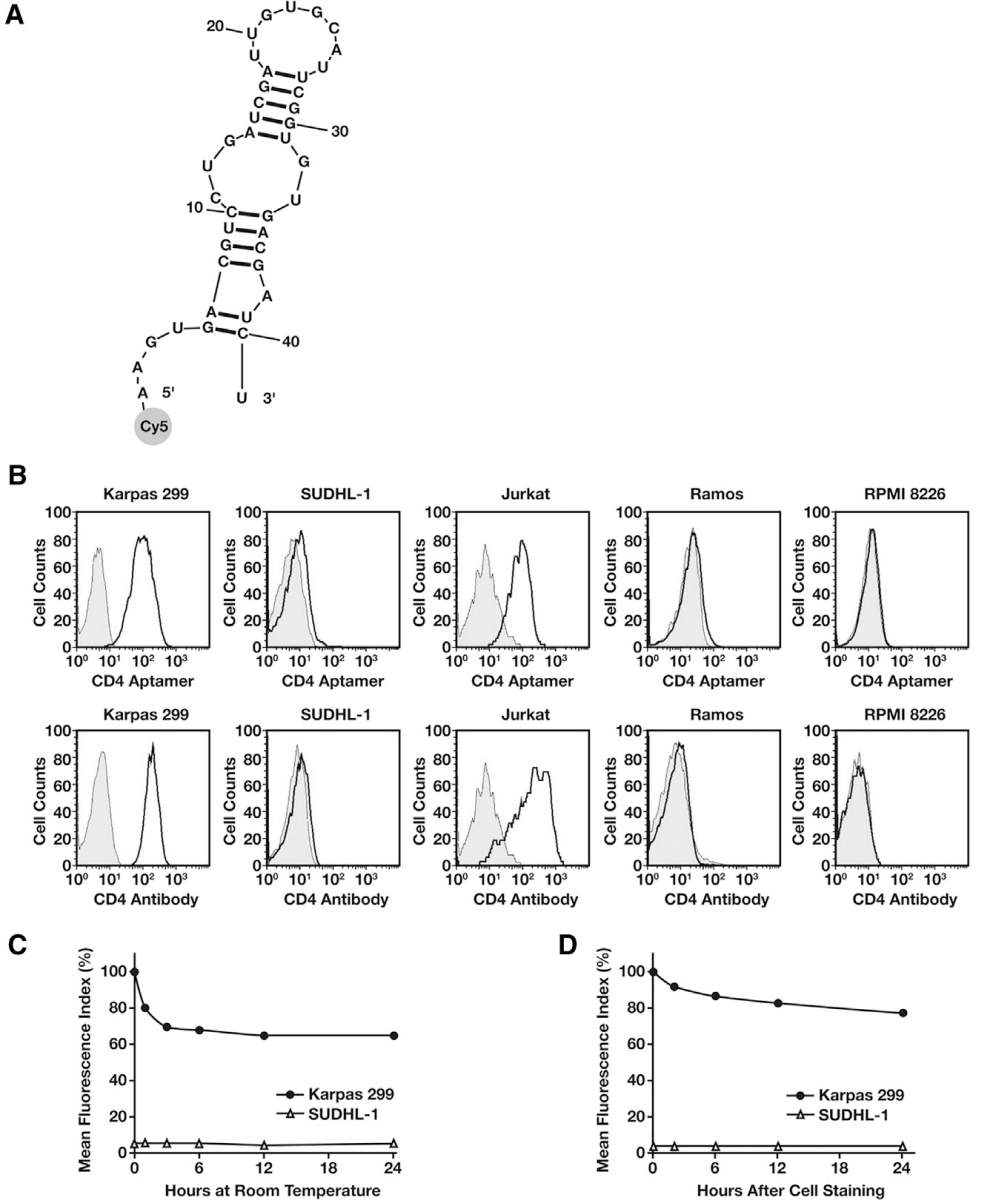
Specific Cell Binding by the Synthetic CD4 Aptamer Probe (A) Sequence and predicted two-dimensional structure of the RNA-based CD4 aptamer probe. A fluorophore reporter (cyanine 5 or Cy5) was conjugated to the 5′ end of the aptamer. (B) Comparison of aptamer and antibody cell-binding patterns, as assessed by flow cytometry. The indicated cells were incubated with the CD4 aptamer (top row) or CD4 antibody as a standard cell-binding control (bottom row). Gray peaks, untreated cells; open peaks, cells incubated with aptamer or antibody. (C) Biostability assay. The residual cell-binding capacity (%) of the CD4 aptamer was quantified by flow cytometry at the indicated times after incubation of the aptamer in cell-binding buffer at room temperature. (D) Biostability assay. The CD4 aptamer was incubated with cells, stored at 4°C for varying times, and then analyzed by flow cytometry. Reproduced from Zhang et al.^20^ with permission.

In 1998, two research groups separately reported the first aptamers, which had high affinity toward CD4 antigen. Using systemic evolution of ligands by exponential enrichment (SELEX) on recombinant rat CD4 molecules, Kraus et al. discovered a set of aptamers that specifically bound to rat CD4 and not to human homologs.^18^ They showed that these aptamers had the ability to inhibit allogeneic mixed lymphocyte reactions by ∼50%. This was the first evidence of modulating immune responses via anti-CD4 aptamers. A month later, Davis et al. reported the first specific RNA aptamers for human CD4 molecules.^19^ They labeled these aptamers with fluorophores and used them in flow cytometry to stain cells expressing CD4 on their surface. These human CD4-specific aptamers selectively stained CD4^+^ T cells in a preparation of human peripheral blood mononuclear cells (PBMCs). The RNA aptamer developed by Davis et al. was later used by Zhang et al. for immunophenotyping.^20^ Flow cytometry confirmed that the aptamer and antibody generated the same CD4 staining pattern without competing with one another (Figure 1). This study also proved that a CD4 aptamer could be used as a substitute for the corresponding antibody in flow cytometry and other cell phenotyping methods.

In 2012, Zhou et al. developed an aptamer-functionalized glass surface using a thiolated RNA aptamer against CD4;^21^ it was based on the original aptamer developed by Davis et al.^19^ Zhou et al. used this platform to capture and enrich CD4-expressing cells. Their results showed more than 6-fold enrichment from a model mixture of CD4^+^ and CD4^−^ cells. Aptamers are favorable candidates for the development of biosensors due to their thermal and physicochemical stability. It is much more convenient to introduce modifications to aptamers than to antibodies. It was suggested that the platform developed by Zhou et al. might be used in new sets of bedside biosensors for diagnosis and HIV/AIDS monitoring.

**Figure 2:**
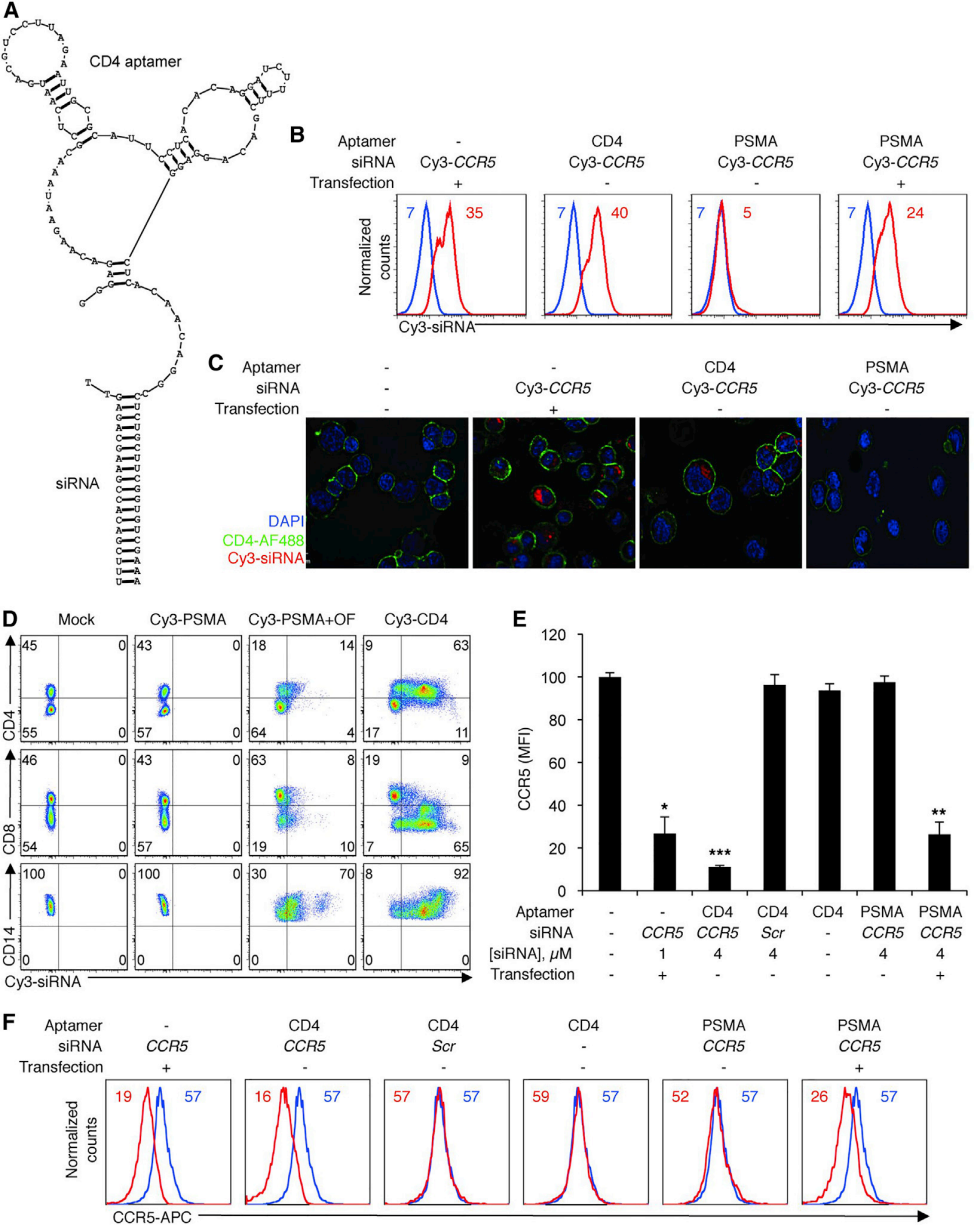
Cy3-Labeled CD4-AsiCs Are Internalized by CD4^+^ Cells and Silence CCR5 Expression In Vitro (A) Design of CD4-AsiC, containing a CD4 aptamer and a CCR5 siRNA. (B and C) CD4-AsiCs or PSMA-AsiCs targeting CCR5 were Cy3 labeled at the 3′ end of the antisense siRNA strand and incubated with primary CD4^+^ T lymphocytes from a healthy donor. Uptake was assessed 24 hr later by flow cytometry (B) and fluorescence microscopy (C; original magnification ×60). Data are representative of three independent experiments. Median fluorescence intensity (MFI) of each peak is shown (mock, blue; treated, red). Transfection controls used nucleofection. (D) Specific siRNA delivery to CD4^+^ cells in a mixed population of resting PBMCs was assessed by flow cytometry 24 hr after incubation with 4 μM Cy3-labeled AsiCs. In the absence of oligofectamine (OF), Cy3-labeled CD4-AsiCs were preferentially taken up by CD3^+^CD4^+^ T cells and CD4^+^CD14^+^ monocytes, whereas PSMA-AsiCs only transfected monocytes with OF. CD3^+^CD8^+^ T cells remained relatively label free. Representative dot plots of three experiments with different donors are shown. (E and F) To test for gene silencing, CD4^+^ T lymphocytes were treated with CD4-AsiCs or PSMA-AsiCs targeting CCR5, with or without transfection. Chimeras bearing scrambled siRNAs (Scr) and CD4 aptamers served as controls. Shown are mean ± SEM. MFI normalized to the mock-treated sample (E; n = 4; *p < 0.005, **p < 0.0005, ***p < 0.00005, two-tailed t test) and representative histograms (F; mock, blue; treated, red). In the absence of nucleofection, CCR5 was knocked down only by CCR5 CD4-AsiCs. Reproduced from Wheeler et al.^23^ with permission.

In 2006, an article in *Nature Biotechnology* entitled “Cell type–specific delivery of siRNAs with aptamer-siRNA chimeras” broadened the applications of aptamers, particularly CD-specific aptamers. In this study, McNamara et al. devised a method for targeted delivery of small interfering RNAs (siRNAs) to prostate cancer cells using prostate-specific membrane antigen (PSMA) aptamers as recognition and internalization agents.^22^ This study was the first evidence of successful functional internalization of aptamer-conjugated siRNAs and consequent gene knockdown. A combination of the studies of McNamara et al. and Davis et al. made an aptamer-siRNA chimera one of the most interesting subjects of research. In 2011, Wheeler et al. showed that CD4 aptamers and siRNA chimeras targeting HIV *gag* and *vif* or host CCR5 were specifically taken up by CD4^+^ cells; and inhibited HIV infection in primary CD4^+^ T cells and macrophages in vitro and in vivo (Figure 2).^23^ They suggested that this cocktail of CD4 aptamers and siRNA chimeras could be used as a topical vaginal microbicide to prevent HIV sexual transmission. Later, in 2013, the same group introduced CD4 aptamer/siRNA chimeras to a hydroxyethylcellulose gel formulation.^24^ Results showed that transmission was completely blocked for 2 days after application in polarized human cervicovaginal explants and humanized mice. In 2012, Zhu et al. again used the original aptamer developed by Davis et al. in the form of a CD4 aptamer-siRNA chimera to inhibit HIV-1 protease expression in T cells.^25^ This time, they converted the reported RNA aptamer to a DNA aptamer to increase the stability of the new chimeric structure. Similar to other previous studies, this CD4 aptamer-siRNA chimera also showed promising results in regards to infection inhibition in vitro. This study also demonstrated that DNA aptamer-based siRNA delivery has inherent advantage in terms of stability.^25^ In the context of siRNA-aptamer chimeras, CD195 (better known as CCR5) has also been used to inhibit HIV infection. CCR5, a protein expressed by T cells and macrophages, is an important co-receptor for HIV-1. Similar to the Wheeler et al. study, the anti-CCR5 aptamer developed by Zhou et al. specifically neutralized virus infection in primary PBMCs and in vivo-generated human CD4^+^ T cells.^26^ Moreover, the CCR5 aptamer was capable of delivering functional anti-HIV siRNAs to CCR5-expressing cells in a receptor-targeted manner.^26^

Following successful reports of delivering siRNAs using CD4 aptamers to helper T cells, Song et al. developed a CD4 aptamer and small hairpin RNA (shRNA) chimera targeting RORγt to suppress Th17 cells.^27^ After successful delivery, RORγt gene expression was suppressed in Karpas 299 cells and CD4^+^ T cells, and consequently, Th17 cell differentiation and interleukin 17 (IL-17) production were inhibited.^27^ Th17 cells and their released cytokines play a critical role in the pathogenesis of autoimmune and inflammatory diseases. Song et al.’s chimeras open a new window for treatment of such diseases due to their desirable targeted effect on Th17 cells.

The newest aptamer developed for CD4 is a single-stranded DNA (ssDNA) discovered by Zhao et al. using cell-SELEX and next-generation sequencing.^28^ Cell-binding assays revealed that this new aptamer had a very high binding affinity for CD4-positive cells and significantly disrupted the viral entry mechanism by displacing viral gp120.

Overall, aptamers against CD4 antigen have demonstrated their capacity in both diagnosis and treatment. They could efficiently replace their antibody rivals and prove to be real substitutes for current antibodies.

### Powerful Immune Response Modulators: CD28, CD137, CD134, CD40, and CD210 Aptamers

According to the three-signal activation hypothesis for T cell activation,^29^ three different types of ligand binding are needed for the proper activation of naive lymphocytes. Besides T cell receptor (TCR) binding and cytokines, the other major signal comes from co-stimulatory molecules.^8^ CD28-B7.2 binding is known to be the main co-stimulatory signal for T cell activation.^8^ With a lack of co-stimulation, lymphocytes enter a stage of anergy and, consequently, T cell tolerance.^30^ For quite some time, anti-CD28 antibodies have been used as artificial co-stimulatory ligands, particularly in the activation of tumor-antigen-specific lymphocytes.^31^, ^32^ Some other humanized antibodies with co-stimulatory capacity, such as antibodies for CD40, OX40, and 4-1BB, are currently in clinical trials.^33^

**Figure 3:**
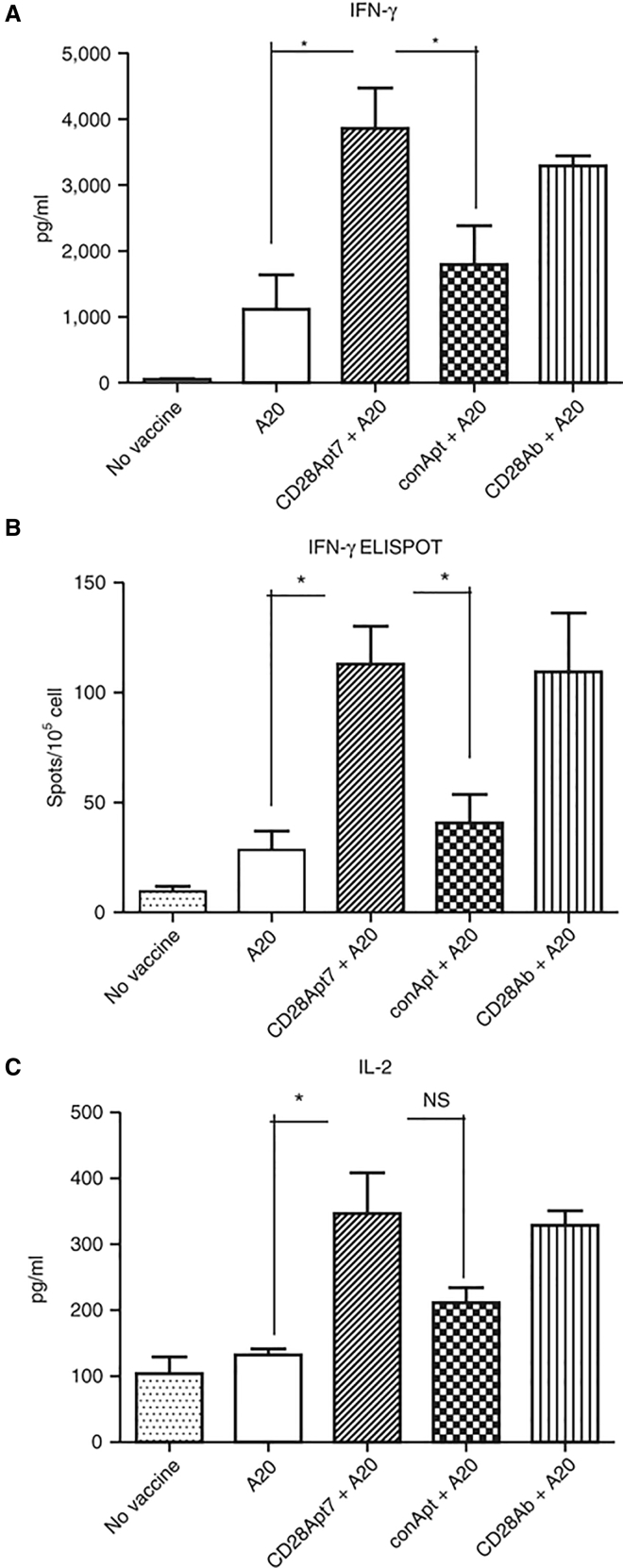
Boosting Cellular Immune Response through CD28Apt7-Dimer (A) Detection of IFN-γ by ELISA from supernatant obtained after a 48 hr co-culture of irradiated A20 cells and lymphocytes obtained from mice previously immunized with irradiated A20 cells plus 400 pmol of the CD28Apt7-dimer, CD28 agonistic antibody 37.51, or a scramble aptamer. The results are expressed as mean and SEM of triplicates. (B) Detection of IFN-γ-producing lymphocytes through ELISPOT after 24 hr co-culture of irradiated A20 cells and lymphocytes obtained from mice previously immunized with irradiated A20 cells plus 400 pmol of the CD28Apt7-dimer, CD28 agonistic antibody 37.51, or a scramble aptamer. The results are expressed as mean and SEM of triplicates. (C) Detection of IL-2 by ELISA from supernatant obtained after a 48 hr co-culture of irradiated A20 cells and lymphocytes obtained from mice previously immunized with irradiated A20 cells plus 400 pmol of the CD28Apt7-dimer, or CD28 agonistic antibody 37.51, or a scramble aptamer. The results are expressed as mean and SEM of triplicates. *p < 0.05. IFN, interferon; IL, interleukin; NS, not significant. Reproduced from Pastor et al.^16^ with permission.

In 2013, Pastor et al.^16^ described two anti-CD28 aptamers showing immunomodulatory capacity. One of these aptamers, CD28Apt2, in its monomeric form reduced the binding of recombinant B7.2 molecules to the CD28 receptor on the surface of HEK293-CD28 cells. The other aptamer, CD28Apt7, did not show a similar effect. For CD28 receptors to trigger the co-stimulation signal, they need to be dimerized.^31^ Based on this fact, Pastor et al. constructed four dimeric aptamer structures from their two developed aptamers. The agonistic effects of these dimeric aptamers on lymphocytes were interesting. Although the CD28Apt7 aptamer did not interfere with ligand binding, its dimeric form promoted the strongest cellular and humoral responses (Figure 3). Pastor et al.’s study showed, for the first time, that a single aptamer could be used as both an agonist and an antagonist. This is a new, relevant feature of aptamers compared to antibodies. In addition, in the same study, the authors showed the potential of the dimeric CD28 aptamer to enhance the anti-tumor effect of the intradermal (Id) vaccine in A20 lymphoma-bearing mice following a vaccination protocol.^16^ In this context, the CD28 aptamer worked as an adjuvant to stimulate a stronger immune response after tumor vaccination. This study showed that CD28 aptamers are strong immune response modulators.

In 2016, Pastor’s group further investigated the potential of their developed CD28 aptamers in two other studies. In Lozano et al.’s study, they used the CD28 aptamer to specifically deliver P60 peptide to CD4^+^ lymphocytes.^34^ P60 is a peptide that can bind to and inhibit Foxp3, a transcription factor that is mostly expressed in T regulatory (Treg) lymphocytes.^35^ Treg lymphocytes have an immunosuppressive phenotype that is used by some tumor cells to evade the immune response.^36^, ^37^ Earlier in this review, we described how CD aptamers can be used to deliver intracellular functional motifs to specific cell populations.^22^ Lazano et al. used the same principle, and the developed CD28 aptamer was used as an internalization agent for P60. The CD28Apt-P60 conjugate successfully entered CD28-expressing cells and reduced Treg immunosuppressive activity in CD4^+^CD25^+^ Treg cells isolated from splenocytes.^34^ This inhibition was more efficient than that produced using unconjugated P60 peptide. The in vivo response of CD28Apt-P60 conjugate was also examined. The enhancing dosage of P60 peptide in ovalbumin-vaccinated mice is ∼5 nmol daily for 5 days. It was shown that a low dose of 250 pmol CD28Apt-P60 conjugate can induce a strong cellular immune response toward ovalbumin, as measured by enzyme-linked immunospot (ELISPOT) and an in vivo killing assay.^34^

In the third study, Pastor’s group constructed a bi-specific aptamer to coat melanoma cancer cells with stimulatory signals for T cells.^38^ As shown in their previous study, dimeric CD28 aptamers were able to trigger a strong co-stimulation signal in antigen-specific T lymphocytes.^16^ They engineered an MRP1-CD28 bivalent aptamer that was able to bind MRP1-expressing tumors and deliver the CD28 co-stimulatory signal to tumor-infiltrating lymphocytes.^38^ Multidrug-resistant-associated protein 1 (MRP1) has been directly correlated with chemotherapy drug resistance in several types of tumors.^39^ In another embodiment of the study, Soldevilla et al. used irradiated tumor cells decorated with MPR1-CD28 aptamer as a vaccine in melanoma-bearing mice and witnessed a tumor size reduction.^38^

These studies clearly demonstrate how a proper aptamer for a key molecule such as CD28 can help us to modulate and manipulate the immune system to our desired extent, from attenuating autoimmunity and graft rejection to treating cancers and, moreover, developing vaccines.

4-1BB (CD137) is another co-stimulatory molecule that has been targeted by antibodies for its role in the survival and expansion of activated T cells.^40^, ^41^ It has been shown that the systemic administration of agonistic anti-4-1BB antibodies enhances tumor immunity and tumor rejection in mice.^33^ The Gilboa team, who previously developed anti-CTLA-4 aptamers, also developed a 4-1BB-binding aptamer.^42^ McNamara et al. showed that bivalent and multivalent configurations of the anti-4-1BB aptamers co-stimulated T cell activation in vitro and promoted tumor rejection in vivo.^42^ Again, this developed aptamer was also used in the context of an siRNA-aptamer chimera to silence mTOR complex 1.^43^ The anti-CD137 aptamer is also very important, since it was the first agonistic aptamer ever reported. Agonistic aptamers are important, since they could be potential substitutes for antibodies, particularly in clinical applications.

OX40 (CD134) is also a co-stimulatory molecule that is expressed by activated T cells.^44^ The signaling pathway, which starts from the OX40:OX40 ligand axis, induces T cell proliferation and cytokine secretion.^44^, ^45^, ^46^ Antibodies have been developed for OX40 that have agonistic effects on T cell activation and are currently in clinical trials as an adjuvant in cancer immunotherapy.^44^, ^45^ Dollins et al. reported the first anti-OX40 RNA aptamers that were generated using SELEX on a recombinant protein.^47^ Scaffold dimerization of this aptamer caused OX40 dimerization in T lymphocytes, which in turn showed increased proliferation, interferon γ (IFN-γ) production, and nuclear translocation of nuclear factor κB (NF-κB) in T cells primed in vivo with staphylococcal enterotoxin B.^47^ Later, in 2013, the same group developed an OX40 aptamer for a human protein.^48^ This RNA aptamer needed to be multimerized to elicit an agonistic effect on activated CD4^+^ T cells. In vitro studies showed that the multimeric OX40 aptamer could induce proliferation and production of IFN-γ in activated CD4^+^ T lymphocytes.^48^

Another immune-stimulatory molecule that has been targeted for its modulatory characteristics is CD40. CD40 is a B lymphocyte surface receptor with several functionalities, including B cell clonal expansion, germinal center formation, isotype switching, affinity maturation, and generation of plasma cells.^49^ CD40 is expressed not only on B cells but also on dendritic cells, macrophages, and hematopoietic precursors.^50^ Due to this broad expression, engagement of CD40 with its ligand, CD40L, may cause various responses, from cell proliferation and anti-tumor effects to mitogenic growth of some tumors, such as B cell lymphomas.^50^, ^51^ In 2015, Soldevilla et al. developed an RNA aptamer for CD40 that could act as both an agonist and an antagonist.^17^ Similar to the study by Pastor et al., dimerization of an antagonistic aptamer created an agonistic CD40 aptamer that could successfully improve recovery from bone marrow aplasia. Additionally, the antagonistic CD40 aptamer effectively blocked the CD40-CD40L interaction and reduced B cell lymphoma proliferation in vitro and in vivo. Moreover, Soldevilla et al. used the developed agonistic CD40 aptamer to construct shRNA-aptamer chimeras to inhibit SMG1, a kinase that is essential for nonsense-mediated mRNA decay (NMD) initiation. Mice treated with the CD40 aptamer-shRNA chimera showed higher tumor infiltration of lymphocytes.^17^

As mentioned earlier, cytokines are one of the sides of the “three-signal activation” hypothesis.^29^ However, cytokines not only participate in immune response activation but also play major roles in immune tolerance, and moreover, some have dual functionality.^52^ Therefore, the ability to manipulate cytokine-induced responses could have a major impact, particularly on immune-related disorders, such as autoimmune disease and cancer. Interleukin 10 (IL-10) is a key immune suppressive agent in multiple pathogenic infections.^53^ Elevated levels of IL-10 are observed in several chronic infectious diseases, including HIV.^53^ There is evidence that anti-IL-10 antibody therapy could improve the condition of mice with lymphocytic choriomeningitis virus.^54^ Also, the role of the IL-10:IL-10 receptor axis is evident in multiple cancers in which target antibody therapy proved to be effective.^55^, ^56^ In 2012, Berezhnoy et al. developed an RNA aptamer specific for the IL-10 receptor (CD210) using SELEX and high-throughput sequencing.^57^ In vitro studies showed that the developed aptamer in tetrameric form could significantly inhibit the IL-10-dependent proliferation of MC/9 cells. Systemic administration of the monomeric form of the aptamer with 2′-*O*-methyl-modified pyrimidines showed a significant reduction in tumor growth in a mouse model after 4 days.^57^ It appears that in co-stimulatory molecules, aptamer-dependent inhibition of cytokine receptors shows significant immune-modulatory effects both in vitro and in vivo. These studies show how CD aptamers can be used directly or indirectly to modulate immune responses, and in cases such as the study by Soldevilla et al., they can be used to correct the devastating side effects of current chemotherapy and radiotherapy treatments.

### Blockade of Immune Checkpoints: CD152, CD279, and CD366 Aptamers

One of the more promising approaches in cancer immunotherapy is the blockade of immune checkpoints.^58^, ^59^ Immune checkpoints are signaling pathways that inhibit immune cells from taking action against particular cell populations that express certain ligands.^58^, ^59^ Immune checkpoints are crucial for maintaining self-tolerance. It has been reported repeatedly that many cancer cells express surface ligands for immune checkpoints to evade immune responses.^58^, ^59^ Due to this means of immune evasion in tumor cells, antibodies that target immune checkpoint components have been shown to significantly induce specific anti-tumor responses.^58^, ^59^ Among these immune checkpoints are three CD molecules that have been widely studied: CTLA-4, PD1, and TIM3.

**Figure 4:**
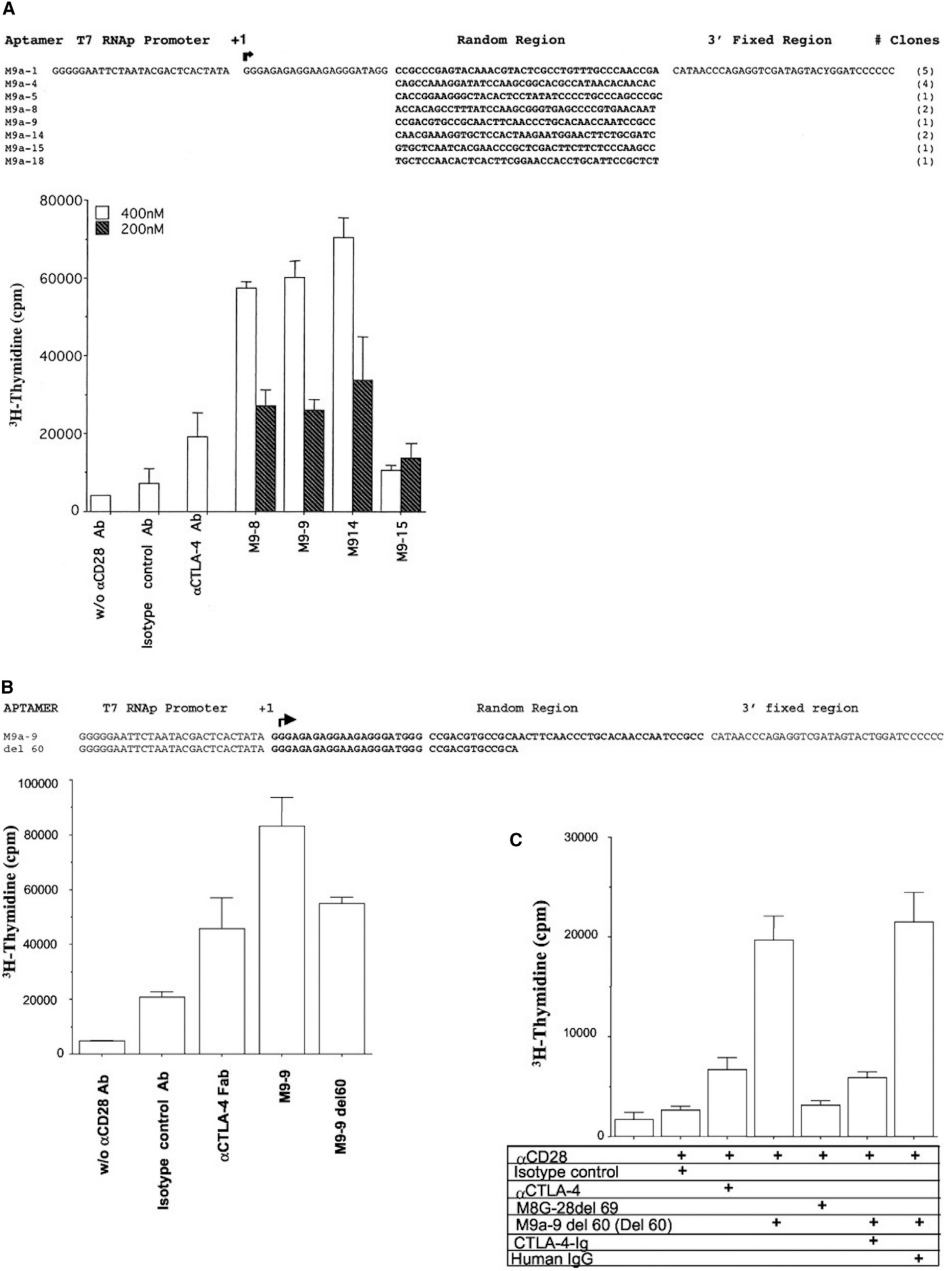
In Vitro Functional Characterization of CTLA-4-Binding Aptamers (A) Sequence of eight clones obtained after nine rounds of selection (M9). The frequency of each clone represented in the M9 pool is indicated in parentheses. In vitro T cell proliferation assays were performed using mouse T cells enriched from lymph nodes stimulated with limiting concentrations of immobilized αCD3 and soluble αCD28 antibody (Ab) in the presence of αCTLA-4 Ab or aptamers. Inhibition of αCTLA-4 function is reflected in increased proliferation of T cells in the presence of Ab or aptamers. Isotype control and αCTLA-4 Ab were used at 20 μg/ml (133 nm) and aptamers at 200 and 400 nm. T cell proliferation was enhanced in the presence of αCTLA-4, but not isotype Ab, and was enhanced in a dose-dependent manner in the presence of M9-8, M9-9, and M9-14, but not M9-15, aptamers. Error bars represent SD. (B) Inhibition of CTLA-4 function in vitro by Del 60, a 36-nt-long synthetic, truncated derivative of M9-9. A model for the predicted secondary structure of the Del 60 aptamer shows the proposed CTLA-4 binding site. A proliferation assay was performed as above except that Fab fragments of isotype control and αCTLA-4 Ab were used at 100 μg/mL (2,000 nm). Error bars represent SD. (C) Inhibition of CTLA-4 with Del 60 and a control aptamer, M8G-28del 69, which binds to CTLA-4 but did not inhibit its function in previous experiments (data not shown). Where indicated, the Del 60 aptamer solution was preincubated with 2-fold excess CTLA-4/Fc or human immunoglobulin G (IgG), followed by protein-G-coated magnetic beads before addition to the T cell cultures. The assay was performed with five replicates per condition. Error bars represent SD. Reproduced from Santulli-Marotto et al.^64^ with permission.

CD152 (widely known as CTLA-4) is an immune checkpoint receptor that is expressed on activated T lymphocytes.^60^ CTLA-4 has a very high affinity for B7 family members and therefore competes with CD28 to bind to these molecules.^61^ Unlike in CD28-B7 binding, the attachment of B7 molecules to CTLA-4 triggers a negative signal, attenuating T cell responses.^61^ Blockade of the function of CTLA-4 using antibodies has shown promising results regarding anti-tumor activity of T cells.^33^, ^61^ There are various studies in mice demonstrating the efficacy of anti-CTLA-4 antibodies in tumor rejection and increased immunity to tumor cells.^62^, ^63^ Based on these promising outcomes, Santulli-Marotto et al. developed RNA aptamers against CTLA-4 to elicit a similar response of tumor rejection after immunotherapy with anti-CTLA4 antibodies (Figure 4).^64^ The developed anti-CTLA4 aptamers inhibited CTLA-4 function in vitro and in vivo. Moreover, the tetrameric form of the anti-CTLA4 aptamers significantly reduced the dosage to elicit anti-tumor responses in vivo. This study showed for the first time that aptamers could be used exactly as antibodies due to their anti-tumor activity in immunotherapy regimens. The same anti-CTLA4 aptamers were also used by Herrmann et al. in the form of a siRNA-aptamer chimera to target tumor-associated CD8^+^ T cells to silence STAT3 and activate tumor-specific T cells.^65^

PD1 or CD279 is another major receptor in immune checkpoints that is expressed on tumor-infiltrating cytotoxic T lymphocytes (CTLs).^66^, ^67^ Engagement of PD1 by its ligand (mostly PD-L1) triggers the phosphorylation of the cytoplasmic tail of PD1, and consequently, the inhibitory signal takes effect in CTLs.^68^ Anti-PD1 monoclonal antibodies have significantly improved immune responses in many different tumor types and have been used as monotherapy or in combination with other agents in more than 50 different trials.^69^, ^70^ Prodeus et al. developed a DNA aptamer that could efficiently block PD1:PD-L1 interaction and therefore suppressed the growth of colon carcinoma cells in vivo.^71^ Prodeus et al. showed that the developed DNA aptamer could elicit the same response as that achieved by blocking the anti-mPD-1 antibody in a murine model.^71^

In 2015, Hervas-Stubbs et al. developed an RNA aptamer for TIM-3, another surface receptor involved in immune checkpoints.^72^ TIM-3 (CD366) is co-expressed with PD1 on many exhausted T cells, but they do not share the same inhibitory mechanism.^73^ There are some studies that show simultaneous blockade of PD1 and TIM-3 has more a significant effect on tumor suppression than on each of them separately.^73^ The monomeric form of the aptamer developed for CD366 by Hervas-Stubbs et al. could significantly activate lymphocytes in vitro and increased IFN-γ secretion.^72^ Combination therapy of the developed aptamer with anti-PD:L1 antibody in a murine model of colon carcinoma also showed synergistic effects on reduction of tumor growth.^72^

These three studies showed that using antagonistic aptamers against immune checkpoints could improve immune responses against tumors. Targeting of immune checkpoints has a particularly important role in inducing immunogenicity of immune-evading tumor cells. These studies show that aptamers can elicit immune responses as effectively as antibodies in various tumor models and that they can be used as enhancers alongside antibodies to trigger more potent immune responses against cancerous tissues.

### Manipulating Cytotoxic Responses: CD16, CD20, CD8, and CD124 Aptamers

So far, we have described how anti-CD antibodies and their potential aptamer counterparts can modulate immune system responses by manipulating signaling pathways. However, one of the pivotal mechanisms in antibody-based therapies is antibody-dependent cellular cytotoxicity (ADCC).^8^ ADCC originates from the interaction of Fc fragments of antibodies with Fc receptors (FcR) on natural killer (NK) cells.^8^ CD16α is an Fc receptor expressed by NK cells, δϒ T cells, monocytes, and macrophages.^74^ In 2011, Boltz et al. developed an aptamer that could elicit a function similar to ADCC, but it was an aptamer-dependent cellular cytotoxicity.^75^ By conjugating the anti-CD16α aptamer to c-Met-specific aptamers, the investigators developed a bi-specific aptamer that targeted the tumor cells in vitro and successfully mediated cellular cytotoxicity dependent on the aptamer and NK cells. This study is another example of how aptamers have the capacity to fully substitute for antibodies for therapeutic reasons.

**Figure 5:**
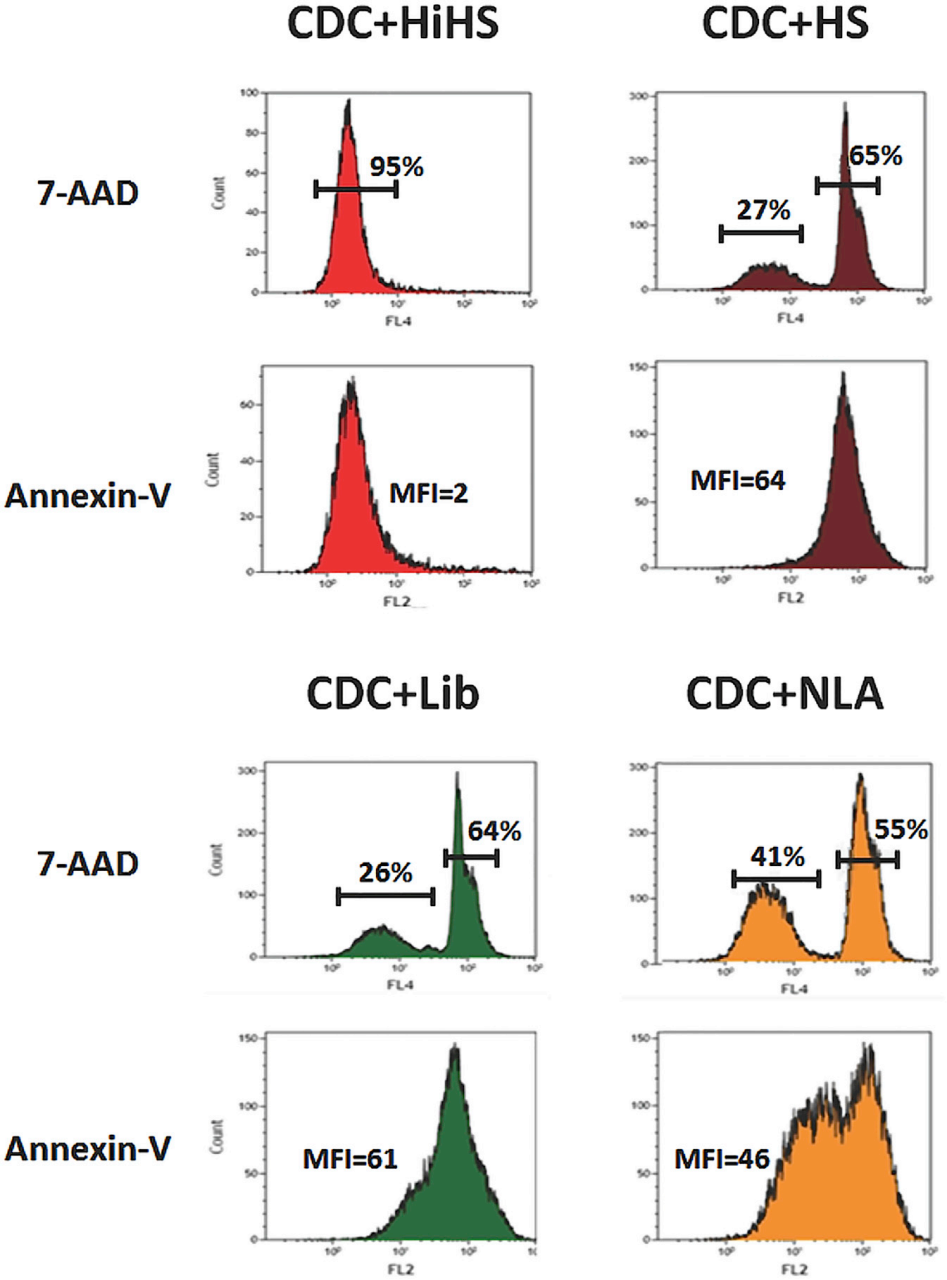
Complement-Dependent Cytotoxicity Was Induced Using 10 ng/μL anti-CD20 Monoclonal Antibody and Viable Human Serum NLA-protected CCL-86 cells exhibit greater overall cellular viability compared to the DNA library (Lib), as indicated by total 7-AAD staining as well as decreased staining of the pro-apoptotic marker Annexin-V. The histograms above represent one result of triplicate samples. MFI, Median fluorescence intensity; HiHS, heat inactivated human serum; HS, human serum; NLA, anti-CD20 aptamer clones. Reproduced from Al-Youssef et al.^76^ with permission.

Aptamers can be used directly to induce cell death and as survival agents to prevent it. The two following aptamers are examples of the latter type. Anti-CD20 aptamers, which were recently developed in our laboratory, efficiently prevented complement-dependent cytotoxicity (CDC) induced by anti-CD20 antibody in B cells.^76^ CD20 is expressed on B lymphocytes and many B lymphomas and is the target of several monoclonal antibodies, such as rituximab, that are used for the treatment of B cell malignancies.^77^ Anti-CD20 antibodies promote ADCC and CDC of B cells expressing CD20 and were found to be very effective at depleting B cells from peripheral blood.^77^ As a limiting agent after the administration of anti-CD20 antibodies, Al-Youssef et al. developed a DNA aptamer that could inhibit CDC by replacing anti-CD20 antibodies (Figure 5).^76^ Inhibiting CDC functionality has other important potential application, since there is evidence for the role of CDC in autoimmune disorders and transplant rejection.^78^, ^79^

Another and more basic way to prevent immune-induced cell death is to inhibit cytotoxic T lymphocytes (CTLs). Wang et al. targeted granulysin (GNLY) in CD8^+^ as a key mediator responsible for the extensive tissue damage seen in patients with autoimmune disorders and graft-versus-host disease (GVHD).^80^ To deliver siRNAs against GNLY, they developed a DNA aptamer that specifically bound to CD8. The constructed a GNLY-siRNA-CD8 aptamer chimera that, like other chimeras mentioned in this review, could specifically target CTLs and silence GNLY expression in drug-induced models of the diseases.

Finally, in regards to the concept that aptamers can elicit cytotoxicity, the anti-IL-4 receptor α (CD124) aptamer is worth mentioning. This aptamer could specifically bind to IL-4Rα expressed on myeloid-derived suppressor cells (MDSCs).^81^ MDSCs have important roles in promoting tumor progression, metastasis, and inhibition of anti-tumor T cell functions.^82^ Upon binding to its target, the anti-CD124 aptamer caused apoptosis in MDSCs and the consequent removal their suppressive effects. In vivo studies showed that the aptamer treatment could significantly delay tumor progression in 4T1-bearing mice.^81^

### Targeting Tumor-Associated Abnormal Expression: CD44 and CD30 Aptamers

The Boltz et al. study is an example of how aptamer constructs can generally target tumor cells regardless of their tumor-specific antigens. Another molecule that is ubiquitously expressed and plays an important role in tumor growth and metastasis is CD44.^83^ CD44 is increasingly recognized as a marker for subpopulations of cancer stem cells (CSCs) that are highly malignant and chemoresistant.^84^, ^85^ The CD44/hyaluronic acid interaction has been investigated in many tumor models, and the results have been promising.^86^, ^87^ Somasunderam et al. developed DNA thioaptamers toward the hyaluronic-acid-binding domain of human CD44.^88^ These aptamers demonstrated a very high affinity for CD44-positive cells. Although the specific function of the developed aptamers as therapeutic agents was not investigated, the investigators provided good material for future studies (e.g., siRNA-aptamer chimeras).

**Figure 6:**
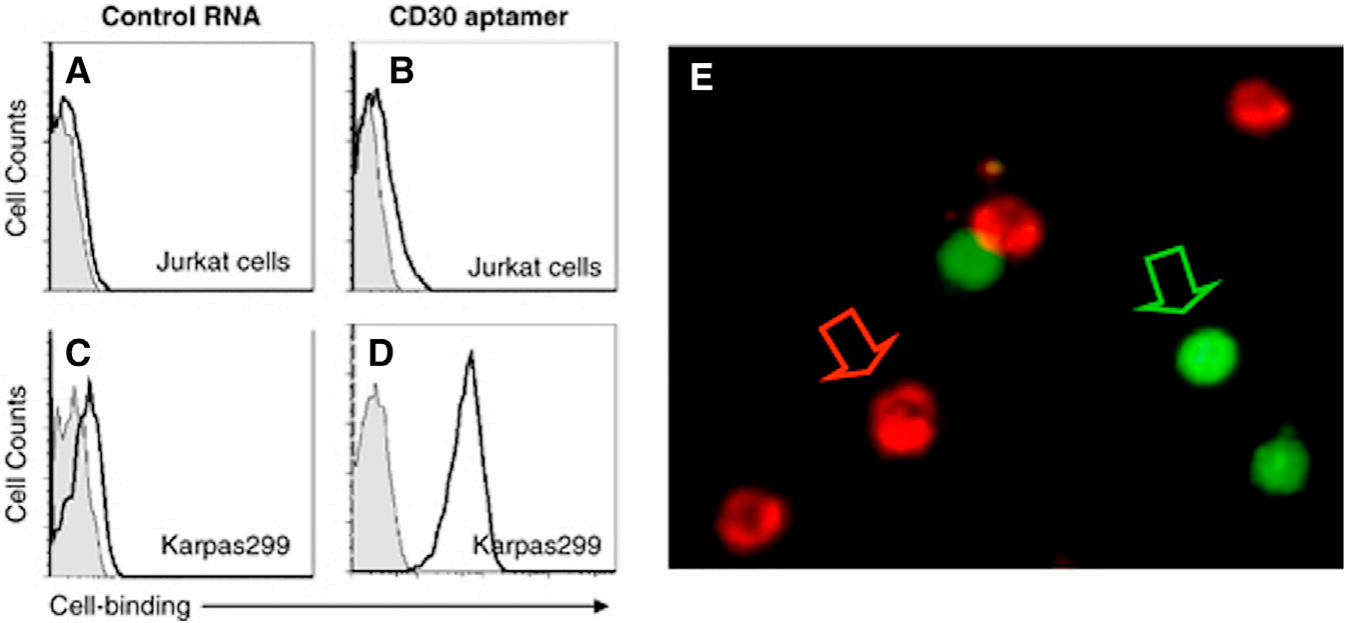
Specific Cell Staining by the CD30 Aptamer (A–D) Cell-binding assays. To rule out nonspecific cell binding, a 39-mer control RNA probe with a random sequence was synthesized and incubated with cultured lymphoma cells. The resultant aptamer-cell binding was examined by flow cytometry. Grey peaks represent unstained cells, and open peaks represent cells that were stained with the aptamer probes as indicated. (E) Fluorescent cell staining by the CD30 aptamer. Jurkat cells were pre-labeled with the fluorescent dye carboxyfluorescein succinimidyl ester (CFSE) and mixed with fresh, untreated Karpas 299 cells. Cell mixtures were then incubated with the Cy5-labeled CD30 aptamer, and the resultant aptamer-cell staining was examined under a fluorescent microscope. Pre-labeled Jurkat cells were identified by green fluorescence and are indicated by the green arrow, and Karpas 299 cells that were specifically stained by CD30 aptamer were identified by red fluorescence and are indicated by the red arrow. Reproduced from Zhang et al.^93^ with permission.

CD30 antigen is the second tumor marker of this kind that has been targeted for aptamer development. Abnormal expression of CD30 has been identified in tumor cells of anaplastic large cell lymphoma (ALCL) and Hodgkin’s lymphoma.^89^ Recently, cellular CD30 receptor targeting represents a new strategy for both diagnosing and treating ALCL.^90^, ^91^ Zhang et al. used developed aptamers that specifically bind to the tumor necrosis factor (TNF) receptor family of proteins^92^ to target CD30 on the surface of ALCL cell lines.^93^ Flow cytometry and fluorescence microscopy studies showed the application of the anti-CD30 aptamer in cell imaging and diagnosis of ALCL (Figure 6). The same group later validated this anti-CD30 aptamer for immunostaining of formalin-fixed and paraffin-embedded tissues.^94^ Like most other CD aptamers mentioned in this review, this CD30 aptamer was also used to create a siRNA-aptamer chimera to target ALCL.^95^ Tumor selectivity and targeted silencing were also the outcome of this study.

### Targeted Delivery: CD268, CD126, and CD205 Aptamers

As mentioned earlier, many of the CD-specific aptamers that have been developed demonstrate internalization abilities. These aptamers have been used in the format of “aptamer-siRNA chimeras” extensively. One of these recently developed aptamers is anti-CD268 aptamer. The expression of B cell-activating factor receptor (BAFF-R or CD268) is restricted on B lymphocytes, and BAFF-R is often overexpressed in B cell malignancies.^96^ The aptamers developed by Zhou et al. efficiently bound to BAFF-R on the surface of B cells, blocked BAFF-mediated B cell proliferation, and were internalized into B cells.^97^ This aptamer was successfully used in the chimeric format with siRNA and silenced the targeted gene, STAT3, in vitro. However, there are other applications for internalizing aptamers, particularly delivering drugs or antigens in a targeted fashion. Anti-CD126 and anti-CD205 aptamers are good examples in this regard.

Interleukin 6 receptor (CD126) is another cytokine receptor that has been targeted for aptamer development. IL-6 is a cytokine that plays a major role in several inflammatory diseases, such as rheumatoid arthritis, Crohn’s disease, and several other cancers, such as myeloma.^98^ It has been shown that IL-6 serves as an anti-apoptotic factor and also a survival factor in certain cancers, such as hepatocellular carcinoma.^99^ Since IL-6 receptor (IL-6R or CD126) is expressed on a few cell types,^100^ in 2012, Meyer et al. targeted the IL-6:IL-6R as a mean of immune manipulation.^101^ Using an RNA aptamer that could specifically recognize IL-6R, they successfully triggered aptamer-receptor-mediated internalization. The RNA aptamer developed by Meyer et al. could bind specifically to both soluble and membrane-associated forms of CD126 without hindering antibody binding.^101^ The ability of this particular aptamer to induce receptor internalization has been shown by cell-specific delivery of fluorescence imaging agents and the streptavidin complex, which were both coupled to the RNA aptamer. In an attempt to turn the developed aptamer into a therapeutic agent, Kruspe et al. replaced the uracil nucleotides in the aptamer structure with 5-fluorouracil, which has been used as an anti-cancer drug for a long time.^102^ Using this genuine approach, Kruspe et al. showed that after specific binding of the RNA aptamer to IL-6R, the receptor-coupled aptamer had been internalized by the cells and lysosomal degradation had mediated the release of 5-fluorouracil, which in turn demonstrated its cytotoxic effects on BaF3 hIL-6R cells.^102^ In another attempt, in 2013, Kruspe et al. conjugated chlorin e6 (ce6) with an IL-6R-binding RNA aptamer.^103^ Ce6 is a photosensitizer molecule that upon excitation releases free radicals. Kruspe et al. demonstrated that light irradiation of cells that had taken up the aptamer-coupled ce6 caused significant cell death.^103^ Free ce6 had no effect on cell population, and cells that had not expressed IL-6R were not affected by this treatment.^103^ Aptamer-induced CD molecule internalization has already been reported, and you may find some examples in the current review. These three studies clearly show how aptamer-induced CD molecule internalization can be effectively used for targeted drug delivery. Recently, Ulrich’s team conducted other studies to expand the usage of their developed aptamer for CD126 to in vivo studies and clinical trials by modifying the aptamer to become more resistant to nucleases.^104^, ^105^

Another interesting use of anti-CD aptamer-targeted delivery is in the development of new vaccines. Wengerter et al. developed a modified RNA aptamer that could specifically bind to CD205 or DEC205, which is predominantly expressed on dendritic cells.^106^ In the presence of the adjuvant polyinosinic:polycytidylic acid (pIC), anti-DEC205 aptamer-ovalbumin conjugates could trigger significant T cell activation in vitro.^106^ These T cells expressed a T cell receptor specific for the major histocompatibility complex I (MHCI)-restricted OVA257–264 peptide SIINFEKL. Further in vivo studies using a flank B16-OVA melanoma model demonstrated that multivalent aptamer-OVA conjugates could specifically induce antigen cross-representation and activate T cells, and consequently, a significant tumor regression was seen.^106^ The findings of Wengerter et al. show the possibility of designing aptamer-based vaccines that can improve targeted delivery of the antigen and also induce cross-representation to achieve better immunity and immune memory.

### Discussion

Currently, the clinical application of aptamers has been limited to a few US Food and Drug Administration (FDA)-approved drugs and a handful of diagnostic kits for some random conditions. Despite numerous successful implementation of aptamers in vitro and in vivo, aptamers have not reached their full extended potential.^13^, ^15^ Some of the core reasons for this are (1) the huge investment in antibody-based diagnostics and therapeutics by pharmaceutical companies; (2) intrinsic biological flaws of unmodified aptamers, such as degradation, considerable renal excretion, non-specific binding, and cross-reactivity; and (3) the “thrombin problem,” which shows the tendency of the research community to improve existing aptamers rather than develop new ones. These problems, alongside others and some solutions, are discussed previously and are cited here.^11^, ^13^, ^14^

Currently, it seems that many issues concerning the biological limitations of aptamers have already been addressed. For example, as seen in Table 1, most researchers have used chemically modified nucleotides to develop aptamers. These modifications are mostly used to improve the intrinsic resistance of the molecules to nuclease attack. Particularly for those aptamers that have been used in in vivo studies, this kind of modification is a prerequisite. Moreover, different studies (here and elsewhere) have used different approaches regarding the SELEX procedure. Yüce et al. reviewed most of these different approaches, explaining their advantages and disadvantages.^107^ Therefore, it seems that aptamer technology has reached its maturation and needs a booster to claim its position. We believe anti-CD aptamers are this booster.

Looking at the abovementioned studies, one should realize the similar path that investigators took to develop applications for their discovered aptamers. Most anti-CD aptamers were first used as cell marker identifier in cell-staining procedures such as flow cytometry and immunohistochemistry. After developing new aptamers with proper affinity, many researchers investigated the developed aptamers for their agonistic and/or antagonistic capacities after binding to CD antigens. This review clearly shows that anti-CD aptamers can strongly manipulate and modulate immune responses through co-stimulatory signals. A simple dimerization can turn an antagonist aptamer into a very powerful agonist; therefore, they can be used separately or together to equilibrate the immune responses. Moreover, anti-CD aptamers can be used to balance the side effects of current therapies, which was described earlier for the anti-CD40 aptamer.^17^

The final stage of development of applications that most researchers followed was to use the anti-CD aptamer in the context of “interfering RNA-aptamer chimeras.” The internalization of aptamers upon binding to their surface targets makes them very efficient carriers, particularly for cells that are not penetrable by other paths. Almost all interfering RNA-aptamer chimeras successfully silenced their target genes in a cell-specific manner. Anti-CD aptamers can open new windows to gene therapy approaches. They can efficiently deliver the genetic material to specific cell populations, from tumor cells to memory T cells and Tregs. Moreover, an antagonist aptamer in the context of interfering RNA-aptamer chimeras can act simultaneously as a blocking agent for the entry of pathogens and, upon internalization, as a treatment for diseased cells. Anti-CD4 and anti-CCR5 aptamers are good examples of this kind of dual-capable aptamers. Furthermore, as Kruspe et al. showed, internalizing aptamers can be turned into potent drugs^102^, ^103^ that can specifically target a particular cell population.

Furthermore, CD antigens are present not only on cells but also on other vesicular entities, particularly extracellular vesicles (EVs). One of the most extensively studied EVs are exosomes, which are small membrane vesicles of endocytic origin that are secreted by most cells in culture and bodily fluids.^108^ They are mostly considered to be a mode of intercellular communication. It is shown that several CD antigens are expressed on exosomes, particularly CD63, CD9, and CD81.^109^, ^110^ These molecules are extensively used as exosome markers to sort and isolate exosomes. Recent studies have shown that tumor cells secrete excessive amounts of exosomes, which have a huge impact on immune system cell responses, particularly as a mean of escape from immune surveillance.^111^, ^112^ There is also evidence that exosomes stimulate immune responses in vivo.^113^ As shown by Zhou et al., anti-CD aptamers can be used to build a platform to capture cells expressing CD antigens on their surface.^21^ The same principle also applies to exosomes. Currently, anti-CD antibodies are used to mark and isolate exosomes. Clearly, anti-CD aptamers can be used as a part of a biosensing device to detect tumor-excreted exosomes in blood or other bodily fluids for early cancer diagnosis or treatment evaluation.

Currently, the use of anti-CD antibodies is a fundamental part of any investigation regarding the immune system, cancer, and studies of emerging pathogens, such as HIV. Anti-CD aptamers can be used as substitutes for antibodies in flow cytometry and other cell phenotyping experiments. Anti-CD aptamers have demonstrated their abilities in several different clinical applications, such as cell phenotyping, immune modulation, cell-targeted delivery, and vaccine adjuvants. Their lower production cost, uniformity, convenient labeling, room temperature stability, and many other advantages of anti-CD aptamers can guarantee their success in this field. Moreover, anti-CD aptamers have shown some capabilities that are unique to aptamers and have not been recognized for antibodies, such as dual-agonist/antagonist functionality and acting as a drug intrinsically.

## Author Contributions

A.N. and M.V.B. wrote the manuscript.

## Conflicts of Interest

The authors declare no competing financial interests.

## Figures and Tables

**Table 1:** Summarized Nucleic Acid Sequences of Anti-CD Aptamers and Their Applications

Target	Species	Type	Aptamer Sequence (5′-3′)	Applications	Reference
CD4	rat	2′-F-RNA^a^	ACUUCAGCUCUAUUAACAGCUCAAGGACUGGCACA	block antibody binding; modulate CD4^+^ cells responses	Kraus et al.^18^
	human	2′-F-RNA^a^	GGGAGACAAGAAUAAACGCUCAAGUGACGUCCUGAUCGAUUGUGCAUUCGGUGUGACGAUCUGGGAGACAAGAAUAAACGCUCAA	cell phenotyping	Davis et al.^19^
			GGGAGACAAGAAUAAACGCUCAAUGACGUCCUUAGAAUUGCGCAUUCCUCACACAGGAUCUUGGGAGACAAGAAUAAACGCUCAA		
			GGGAGACAAGAAUAAACGCUCAAUGUGUCGUCCUGGUACGAUUUUGGUAUAUAACCGUGGCUUGGGAGACAAGAAUAAACGCUCAA		
		5′-Cy5-RNA	AAGUGACGUCCUGAUCGAUUGUGCAUUCGGUGUGACGAUCU	cell phenotyping	Zhang et al.^20^
		5′-thiol- RNA	GUGACGUCCUGAUCGAUUGUGCAUUCGGUGUGACGAUCU	affinity adsorption; biosensors	Zhou et al.^21^
		aptamer - siRNA chimera (2′-F-RNA^a^)	UGACGUCCUUAGAAUUGCGCAUUCCUCACACAGGAUCUU	cell-specific siRNA cell delivery; inhibiting HIV infection in vitro and in vivo	Wheeler et al.^23^
			GUGACGUCCUGAUCGAUUGUGCAUUCGGUGUGACGAUCU		
		DNA aptamer- siRNA chimera	GTGACGTCCTGATCGATTGTGCATTCGGTGTGACGATCT	inhibiting HIV infection in vitro	Zhu et al.^25^
		aptamer - shRNA chimera (2′-F-RNA^a^)	GGGAGACAAGAAUAAACGCUCAAUGACGUCCUUAGAAUUGCGCAUUCCUCACACAGGAUCUUUUCGACAGGAGGCUCACAACAGGC	cell-specific shRNA delivery; inhibiting Th17 cell differentiation and IL-17 production in vitro	Song et al.^27^
		DNA	ATCCAGAGTGACGCAGCACCACCACCGTACAATTCGCTTTCTTTTTTCATTACCTACTCTGGC	cell phenotyping; blocking the interaction of HIV gp120	Zhao et al.^28^
			CCACCACCGTACAATTCGCTTTCTTTTTTCATTACCTACTCTGGCTGGACACGGTGGCTTAGT		
CD8	human	DNA aptamer-siRNA chimera	CTACAGCTTGCTATGCTCCCCTTGGGGTA	targeted siRNA delivery; inhibiting cytotoxic T-lymphocyte-mediated drug hypersensitivity	Wang et al.^80^
CD16	human	DNA	CCACTGCGGGGGTCTATACGTGAGGAAGAAGTGG	aptamer-dependent cellular cytotoxicity	Boltz et al.^75^
CD20	human	DNA	CTCCTCTGACTGTAACCACGGCACGTACGAAACGCATGAGTGCGGACATCCACGCGGCGCGCTCACATGGCTATGTGTACGCATAGGTAGTCCAGAAGCC	inhibition of complement-dependent cytotoxicity	Al-Youssef et al.^76^
			CTCCTCTGACTGTAACCACGCTGCCCACTCCACATGCCTGCGCCGTCAATCACTTCATGCACGCTCGCGTTTACCCGTATGCATAGGTAGTCCAGAAGCC		
			CTCCTCTGACTGTAACCACGCCGTATGTCCGAAATACGGAGAACAGCACTCATATGCAAGCCATACGCGGAGGTGCACGCGCATAGGTAGTCCAGAAGCC		
			CTCCTCTGACTGTAACCACGACACACGGAGGGCATGTGCACGAAGATACATGGGCGTAACATGCTTGCCGCATCGCGCGTGCATAGGTAGTCCAGAAGCC		
CD28	human	2′-F-RNA^a^	GGGAGAGAGGAAGAGGGAUGGGGAUUAGACCAUAGGCUCCCAACCCCCAUAACCCAGAGGUCGAUAGUACUGGAUCC	CD28 antagonist; enhancement of anti-tumor vaccines	Pastor et al.^16^
				peptide delivery; inhibiting Treg function in vitro and in vivo	Lozano et al.^34^
				delivering T lymphocytes to tumor cells by MRP1-CD28 bi-specific aptamer	Soldevilla et al.^38^
CD30	mouse/human	RNA^b^	gauUCGUAUGGGUGGGAUCGGGAAGGG CUACGAACAccg	diagnosis of anaplastic large cell lymphoma (ALCL); tissue labeling	Zhang et al.^93^
CD40	mouse	aptamer (2′-F-RNA^a^) - shRNA chimera	GGGAGAGACGAUGCGGCCAACGAGUAGGCGAUAGCGCGUGGCAGAGCGUCGCUGAGGAUCCGAGA	blocking or activating CD40; inhibiting nonsense-mediated mRNA decay by the aptamer-shRNA chimera	Soldevilla et al.^17^
CD44	human	DNA^c^	CCAAGGCCTGCAAGGGAACCAAGGACACAG	cell targeting	Somasunderam et al.^88^
			TTGGGACGGTGTTAAACGAAAGGGGACGAC		
CD124 (IL4Rα)	mouse	RNA	AAAAAGCAACAGGGGUGCUCCAUGCCGCAUGGAACCUCCGCG	cancer immunotherapy	Roth et al.^81^
CD126 (IL6R)	human	RNA	GGGGAGGCUGUGGUGAGGG	it is internalized and delivers a protein	Meyer et al.^101^
CD134 (OX40)	mouse	2′-F-RNA^a^	GGGAGGACGAUGCGGCAGUCUGCAUCGUAGGAAUCGCCACCGUAUACUUUCCCACCAGACGACUCGCUGAGGAUCCGAGA	receptor agonist; cell-based tumor vaccines	Dollins et al.^47^
	human	2′-F-RNA^a^	GGGAUGCGGAAAAAAGAACACUUCCGAUUAGGGCCCACCCUAACGGCCGCAGAC		Pratico et al.^48^
CD137 (4-1BB)	mouse	2′-F-RNA^a^	GGGAGAGAGGAAGAGGGAUGGGCGACCGAACGUGCCCUUCAAAGCCGUUCACUAACCAGUGGCAUAACCCAGAGGUCGAUAGUACUGGAUCCCCCC	cell-specific siRNA delivery; T cell activation in vitro; tumor rejection in vivo	McNamara II et al.^42^
CD152 (CTLA-4)	mouse	2′-F-RNA^a^	GGGAGAGAGGAAGAGGGAUGGGCCGACGUGCCGCA	inhibiting CTLA-4 function in vitro and in vivo	Santulli-Marotto et al.^64^
		aptamer (2′-F-RNA^a^) - siRNA chimera		cell specific siRNA delivery; Activating tumor specific T cells	Herrmann et al.^65^
CD195 (CCR5)	human	aptamer (2′-F-RNA^a^) - siRNA chimera	GGGAGGACGAUGCGGGCCUUCGUUUGUUUCGUCCACAGACGACUCGCCCGA	cell-specific siRNA delivery; inhibiting HIV infection in vitro and in vivo	Zhou et al.^26^
CD205 (DEC 205)	mouse	2′-F-RNA^a^	GGGAGGUGUGUUAGCACACGAUUCAUAAUCAGCUACCCUCCC	delivers an antigen to dendritic cells, results in T cell activation	Wengerter et al.^106^
CD210 (IL10R)	mouse	2′-F/OMe-RNA^a^^,^^b^	GGGCUCCUGUAAUUGGcGUAUGUAAcCCAGGCAcCAAAcACCCCCCUU	blockade of IL-10 receptor; cancer therapy	Berezhnoy et al.^57^
CD268 (BAFF-R)	human	aptamer (2′-F-RNA^a^)/ siRNA chimera	GGGAGGACGAUGCGGGAGGCUCAACAAUGAUAGAGCCCGCAAUGUUGAUAGUUGUGCCCAGUCUGCAGACGACUCGCCCGA	blocks B cell proliferation and delivers siRNA	Zhou et al.^97^
CD279 (PD1)	mouse	DNA	GACGATAGCGGTGACGGCACAGACGGCTACTGTACATCACGCCTCTCCCC CGTATGCCGCTTCCGTCCGTCGCTC	inhibiting the PD1:PD-L1 inhibitory axis in cytotoxic T lymphocytes	Prodeus et al.^71^
			GACGATAGCGGTGACGGCACAGACGGTACAGTTCCCGTCCCTGCACTACA CGTATGCCGCTTCCGTCCGTCGCTC		
CD366 (TIM-3)	mouse	2′-F-RNA^a^	GGGAGAGGACCAUGUAGUCACUAUGGUCUUGGAGCUAGCGGCAGAGCGUCGCGGUCCCUCCC	receptor antagonist; cancer therapy	Hervas-Stubbs et al.^72^

Underlined nucleotide sequences are PCR primer adaptor sequences.

a 2′-F-pyrimidine-containing RNA.

b Lowercase nucleotides contain 2′-*O*-methyl modifications.

c The anti-CD44 aptamers contain monothiophosphate substitutions (in the *S*_p_ configuration) on the 5′ side of every dA residue.
